# Mediating Effect of Social Engagement Between Neighborhood Environment and Cognitive Dysfunction

**DOI:** 10.1093/geroni/igaf122.2906

**Published:** 2025-12-31

**Authors:** Min Kyung Park, Bada Kang, Chang Won Won, Dahye Hong, Seok-Jae Heo, Chang Gi Park, Miji Kim, Eleanor McConnell

**Affiliations:** Yonsei University College of Nursing, Seoul, Republic of Korea; Yonsei University College of Nursing, Seoul, Republic of Korea; Kyung Hee University, Seoul, Republic of Korea; Yonsei University College of Nursing, Seoul, Republic of Korea; Yonsei University College of Nursing, Seoul, Republic of Korea; University of Illinois Chicago, Chicago, Illinois, United States; Kyung Hee University College of Medicine, Seoul, Republic of Korea; Duke University, Durham, North Carolina, United States

## Abstract

Cognitive dysfunction among older adults is a significant health and social issue worldwide; however, previous studies have primarily focused on individual-level risk factors. Therefore, this study examined the longitudinal relationship between older adults’ neighborhood environment and cognitive dysfunction and investigated the mediating role of social engagement in this relationship. Using data from the Korean Frailty and Aging Cohort Study (2016–2023), we analyzed 1,262 community-dwelling older adults aged 70–84 years. Cognitive dysfunction was measured based on the number of cognitive tests results in a seven-test battery that were below the cutoff point. The neighborhood environment was assessed using the Perceived Neighborhood Environment Scale, used to evaluate physical and social aspects of a residential area. Social engagement was measured based on participation in eight types of social gatherings or group activities. To examine the longitudinal relationship and mediation effect, we used linear mixed-effects models and Sobel’s test. At baseline, the participants had an average age of 76.37 years, 51% were women, and 955 individuals (75.7%) exhibited cognitive dysfunction on at least one cognitive test. Access to destinations had a statistically significant negative relationship with the extent of cognitive dysfunction (β=-0.056, p = <.001), with social engagement partially mediating this relationship (mediation effect: β=-0.002, p<.001). Moreover, aesthetic quality also had a negative relationship with the extent of cognitive dysfunction; however, social engagement did not significantly mediate this relationship. These findings highlight the importance of community interventions aiming to improve neighborhood accessibility and encourage social engagement for supporting cognitive well-being in older adults.

